# Evidence and Perspectives for Choline Supplementation during Parenteral Nutrition—A Narrative Review

**DOI:** 10.3390/nu16121873

**Published:** 2024-06-14

**Authors:** Wolfgang Bernhard, Katrin A. Böckmann, Michaela Minarski, Cornelia Wiechers, Annegret Busch, Daniela Bach, Christian F. Poets, Axel R. Franz

**Affiliations:** 1 Department of Neonatology, University Children’s Hospital, 72076 Tübingen, Germany; wolfgang.bernhard@med.uni-tuebingen.de (W.B.); katrin.boeckmann@med.uni-tuebingen.de (K.A.B.); michaela.minarski@med.uni-tuebingen.de (M.M.); cornelia.wiechers@med.uni-tuebingen.de (C.W.); christian-f.poets@med.uni-tuebingen.de (C.F.P.); 2 Pharmaceutical Department, University Hospital, 72076 Tübingen, Germany; annegret.busch@med.uni-tuebingen.de (A.B.); daniela.bach@med.uni-tuebingen.de (D.B.); 3 Center for Pediatric Clinical Studies, University Children’s Hospital, 72076 Tübingen, Germany

**Keywords:** betaine, choline, cholestasis, hepatosteatosis, liver disease, methyl donors, parenchymal integrity, parenteral nutrition, phosphatidylcholine, preterm infants

## Abstract

Choline is an essential nutrient, with high requirements during fetal and postnatal growth. Tissue concentrations of total choline are tightly regulated, requiring an increase in its pool size proportional to growth. Phosphatidylcholine and sphingomyelin, containing a choline headgroup, are constitutive membrane phospholipids, accounting for >85% of total choline, indicating that choline requirements are particularly high during growth. Daily phosphatidylcholine secretion via bile for lipid digestion and very low-density lipoproteins for plasma transport of arachidonic and docosahexaenoic acid to other organs exceed 50% of its hepatic pool. Moreover, phosphatidylcholine is required for converting pro-apoptotic ceramides to sphingomyelin, while choline is the source of betaine as a methyl donor for creatine synthesis, DNA methylation/repair and kidney function. Interrupted choline supply, as during current total parenteral nutrition (TPN), causes a rapid drop in plasma choline concentration and accumulating deficit. The American Society for Parenteral and Enteral Nutrition (A.S.P.E.N.) defined choline as critical to all infants requiring TPN, claiming its inclusion in parenteral feeding regimes. We performed a systematic literature search in Pubmed with the terms “choline” and “parenteral nutrition”, resulting in 47 relevant publications. Their results, together with cross-references, are discussed. While studies on parenteral choline administration in neonates and older children are lacking, preclinical and observational studies, as well as small randomized controlled trials in adults, suggest choline deficiency as a major contributor to acute and chronic TPN-associated liver disease, and the safety and efficacy of parenteral choline administration for its prevention. Hence, we call for choline formulations suitable to be added to TPN solutions and clinical trials to study their efficacy, particularly in growing children including preterm infants.

## 1. Introduction

Preterm delivery before 37 completed weeks postmenstrual age (PMA) accounts for ~10% of all births worldwide, with 1–2% neonates being born as very low (<1500 g; VLBWI) or extremely low (<1000 g; ELBWI) birth weight infants [1,2,3,4]. Prematurity is the largest single contributor to life-years spent in disability, and cognitive impairment is common compared to term born children even in the absence of brain lesions [5]. In spite of sufficient supply with energy, protein and other macro- and micronutrients with current enteral and parenteral nutrition, growth and neurocognitive development of preterm infants is particularly poor following gastrointestinal complications such as focal intestinal perforation (FIP) and necrotizing enterocolitis (NEC), i.e., in infants who require prolonged periods of total (or partial) parenteral nutrition (TPN) [6].

Current estimates suggest a need for a higher supply with additional nutrients. While arachidonic acid (ARA) and docosahexaenoic acid (DHA) are under discussion for their impact on long-term outcome [7], choline is similarly suggested as a critical nutrient for preterm infant development, being tightly linked to liver function, lipoprotein metabolism, cognitive development and ARA/DHA metabolism. Choline is a constituent of phosphatidylcholine (PC), the major phospholipid of all membranes and of many secretions, like lipoproteins and bile, with rapid hepatic turnover. The cellular uptake of choline, and consequently PC synthesis, is proportional to the plasma concentration of choline, due to the high constant of Michaelis of ubiquitous choline transporters [8,9]. PC is also required to synthesize sphingomyelin (SPH), the membrane phospholipid with the second-highest amount of choline, from (pro-apoptotic) ceramides. High SPH synthesis from PC occurs particularly in the lungs [10]. In all mammals, large amounts (~40%) of both enterally and parenterally administered choline are oxidized to betaine, resulting in a substantial increase in betaine concentrations upon choline administration [10,11,12,13,14]. As a methyl donor for the synthesis of S-adenosyl-methionine (SAM), betaine is required for epigenetic control, creatine synthesis, endogenous PC synthesis from phosphatidylethanolamine (PE) by PE-N-methyltransferase (PEMT) and many other methylations [13,15]. Notably, this pathway is linked to exogenous choline and contributes little to choline synthesis in infants [14]. The short plasma half-life time of choline (<1 h), caused by cellular uptake and rapid metabolism to PC and betaine, suggests a need for continuous infusion during TPN to achieve constant physiologic concentrations [5,15,16,17]. In essence, high total choline concentrations are present in all parenchymal tissues [15], but low postnatal choline supply not meeting the presumed requirements and subsequently low plasma concentrations [18], together with decreased lean body mass growth at and beyond term equivalent age [19] suggest that choline may be growth limiting in preterm infants.

Despite recognition as an essential nutrient by the US Institute of Medicine (IoM) and European Food Safety Authority (EFSA) [20,21,22], the need for choline during parenteral nutrition is not yet generally acknowledged by all relevant medical societies [23,24,25,26]. 

A requirement of choline supplementation during TPN is consistent with several preclinical and a limited number of clinical observational and interventional studies. Data suggest even higher choline requirements in children, particularly preterm infants, compared to adults, where choline deficiency is a major contributor to parenteral nutrition-associated liver disease (PNALD). For adults, data indicate benefits from both preventive and therapeutic choline supply during TPN. We therefore performed this narrative review based on a systematic literature search on choline during TPN to draw attention to this issue particularly for growing pediatric TPN patients, including preterm infants.

## 2. Materials and Methods

To review current knowledge on the impact of choline deficiency and supplementation in patients receiving TPN, we performed a literature search in Pubmed on 23 January 2023 (with an update on 27 April 2024) with the search strategy “choline” and “parenteral nutrition”. Titles were scanned for relevance to the topic by the authors and potentially related publications evaluated as full text. Additionally, reference lists of these publications and forthcoming statements of organizations like EFSA (see below) were searched. Interventional studies involving animals or humans, and other studies that require ethical approval, had to list the authority that provided approval and the corresponding ethical approval code.

## 3. Results

**Figure 1:**
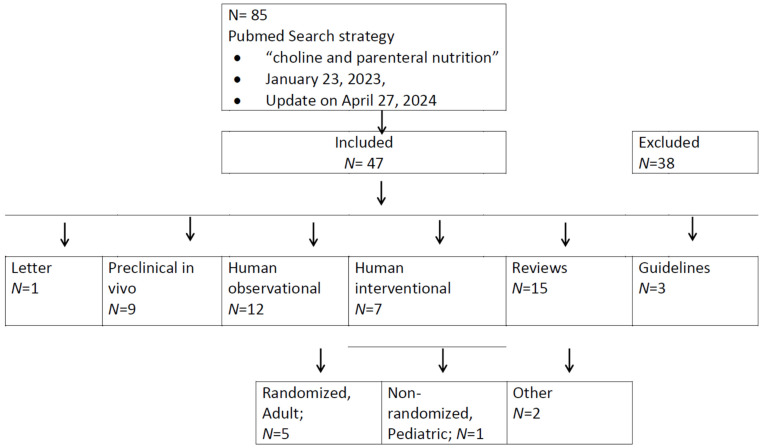
Flow diagram of PubMed search for choline in parenteral nutrition. Please refer to the text for references.

The primary literature search retrieved 84 publications, with an additional one found at the update [27] (see flow diagram, Figure 1). Upon evaluation of their titles, 38 were considered of no relevance (for complete search results see Supplementary Table S1). The remaining 47 articles were evaluated in more detail, including a letter pointing to the evidence for choline deficiency in TPN [28], followed by 9 pre-clinical in vivo studies (1985–2020) [29,30,31,32,33,34,35,36,37], 20 human trials (12 observational (1985–2021) [18,38,39,40,41,42,43,44,45,46,47,48] and 7 interventional studies (1990–2011) [49,50,51,52,53,54,55,56] including one editorial [51] on ref. [50]), 14 reviews (1986–2019) [15,57,58,59,60,61,62,63,64,65,66,67,68] plus 1 in 2024 [27] and 3 guidelines (2012–2022) [26,69,70]. Of the interventional studies, five were randomized addressing adult patients, only one non-randomized study included pediatric patients [56].

### 3.1. A Letter to the Editor

In 1980, Burt and colleagues reported a decrease in plasma choline concentration from 7–9 µmol/L to unphysiologically low levels of 2–4 µmol/L in response to TPN in adult patients within 14 d. Choline levels at the start of TPN were not different from those of other patients, and the decrease was observed irrespective of initial individual concentrations [28].

### 3.2. Preclinical Studies

Based on the frequent development of steatosis and cholestasis in TPN patients, particularly children [71], Hall and colleagues [29] randomized adult rats to TPN with or without choline hydrogen citrate and found no effect on hepatic triglyceride content or secretion. However, the reported choline dose of 27 mg choline hydrogen citrate/kg body weight per day was rather low (see below).

Parenteral administration of 4 mL/kg/d choline-free Intralipid^®^ 20% (Fresenius Kabi, Lake Zurich, IL, USA) to adult New Zealand rabbits for 15 d was found to be critical for steatosis development. Intravenous (IV) and intraperitoneal (IP) administration resulted in increased plasma triglycerides, cholesterol and phospholipids, whereas no changes compared to controls developed after intragastric administration [30]. Only the IV group showed increased hepatic triglycerides and a 5–6-fold decrease in the phospholipid to triglyceride ratio in plasma.

**Figure 2:**
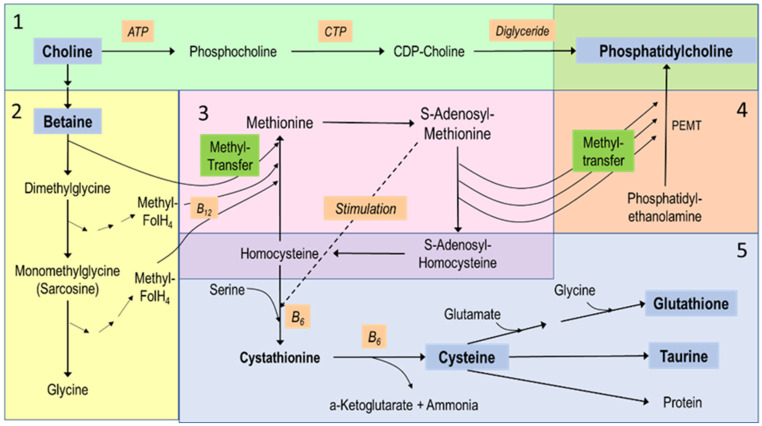
Choline metabolism and its relations to other nutrients. The figure reflects major hepatic pathways and is divided into 5 sections: (**1**) (green) the synthesis of phosphatidylcholine (PC) de novo via the Kennedy pathway, using exogenous choline and requiring adenosine triphosphate (ATP), cytidine triphosphate (CTP) for cytidinediphosphocholine (CDP choline) formation and diglyceride as the acceptor of phosphocholine. (**2**) The downstream metabolites of choline, starting with its 2-step oxidation to betaine, followed by sequential demethylation to provide methyl groups for the synthesis of methionine from homocysteine. (**3**) The methionine-homocysteine circle, where methionine is subsequently activated to S-adenosylmethionine that provides methyl groups for nearly all methylation processes. (**4**) One example of such methylations, i.e., the synthesis of phosphatidylcholine from phosphatidylethanolamine (PE) by 3-fold sequential methylation requiring PE-N-methyltransferase (PEMT). Here, the highly unsaturated fatty acyl residues of PE rather than of diglyceride pools (see **1**) define the molecular composition of PC. (**5**) The hepatic transsulfuration pathway and its link to the methionine-homocysteine circle (**3**) that is fed by choline’s methyl groups (**2**). This pathway serves the synthesis of cysteine via cystathionine as an intermediate—which is stimulated by high S-adenosylmethionine concentrations. These reactions require vitamin B6 (B6), while cysteine then serves taurine and glutathione synthesis.

In term-born, 15–18 d old, milk-fed healthy rats, cysteine was critical for the prevention of hepatosteatosis and normalization of plasma lipids in response to TPN feeding [32]. In line with this finding, and the close link between the metabolism of cysteine, choline and SAM [72,73] (Figure 2), Oz and coworkers demonstrated that administration of SAM or the cysteine precursor N-acetyl-4-thiazolidine carboxylic acid significantly improved liver steatosis, decreased elevated transaminases in plasma and reduced the expression of pro-fibrotic genes [33].

In 3 weeks old healthy rats, 7 d of choline-free TPN increased the generation of reactive oxygen species (ROS) and tumor necrosis factor alpha (TNFα), whereas plasma choline and antioxidant capacity decreased. Choline-free TPN in rats caused liver pathology and steatosis, increased plasma transaminases, triglycerides, very low-density lipoproteins (VLDL), bile acids and bilirubin as well as peroxisomal proliferator-activated receptor alpha (PPARα) gene promoter methylation, which were reversed by 600 mg/kg/d IV choline chloride (450 mg/kg/d choline) [34,35].

Because choline deficiency, caused by TPN, may contribute to small intestinal disease and atrophy, olive oil-based parenteral fat emulsion, with and without parenteral choline, was administered to 3 weeks old rats showing that 450 mg/kg/d IV choline ameliorated enterocyte apoptosis and mitochondrial dysfunction and tended to increase villous height. [36]. 

In a PNALD model involving 3 weeks old rats, 7 days of choline-free TPN [37] decreased PC synthesis, changed composition of conjugated bile acids, decreased riboflavin (vitamin B2) and long-chain polyunsaturated fatty acids (LC-PUFA), and blocked acetyl-coenzyme A formation from pyruvate.

In summary, preclinical studies suggest an adequate choline status, together with that of the choline-related compounds cysteine and SAM, as critical to hepatic lipid homeostasis during TPN. They suggest that deficiency in choline, together with that of sulfur-containing compounds (cysteine, taurine), riboflavin (vitamin B2), methyl donors (SAM), and LC-PUFA, contribute to PNALD. A summary of the metabolic relations between choline and related metabolites is shown in Figure 2.

### 3.3. Observational Studies in Humans

In undernourished adult (14–52 years old) TPN patients fed parenteral amino acids and other water-soluble nutrients, plasma levels of free choline, taurine, cysteine, glutathione, protein-bound cysteine and phosphatidylcholine were decreased, whereas methionine was elevated [38]. 

In undernourished adults, plasma choline was lower compared to healthy individuals (6.5 ± 0.6 µmol/L vs. 9.7 ± 0.7 µmol/L) at baseline and continued to decrease to 4.7 ± 0.5 µmol/L after 1 week of TPN [39]. Plasma choline recovered to its initial level (6.8 ± 0.9 µmol/L), but not to normal values following parenteral lipid infusion supplemented with as little as 0.7 mg/d free choline and 240 mg/d choline equivalent as PC (i.e., ~44% of AI values), given orally in combination with incremental enteral feeding up to 1000 kcal/d [20]. 

Buchman et al. [40] reported low choline plasma concentrations in 41 TPN patients (0.1 to 79 years). This also applied to the seven children included, five of whom were below one year. Patients received only minute amounts of free choline (24 ± 6 nmol/mL = 2.5 µg/mL), but 11.63 ± 0.55 µmol/mL lipid-bound choline (=1.6 mg/mL choline equivalent) via parenteral lipid emulsions. At a supply of 22.4 ± 7.1 kcal/kg/d via TPN, this equals 28 ± 9 µg/kg/d of free choline (~0.5% of AI) and 18 ± 6 mg/kg/d phospholipid-bound choline (~twice the AI) [40]. Choline concentrations varied widely (3.3–15.6 µmol/L), with subnormal values in 80% of patients, and the mean concentration (7.2 ± 2.5 µmol/L) being below that of fasting adult control levels (11.3 ± 3.7 µmol/L). Serum alanine aminotransferase (ALT) and aspartate aminotransferase (AST) were increased and correlated inversely with plasma concentrations of free choline. In essence, IV supply of lipid-bound choline (as PC), although exceeding the AI, was unable to normalize free choline concentrations in plasma (as already shown [39]) or to prevent PNALD.

Additionally, 16 pediatric TPN patients (3 to 47 months) with severe gastro-intestinal disorders and duodeno-gastric reflux showed continuous enteral (free) choline secretion (0.128–0.271 µmol/kg/h = 13–28 µg/kg/h), suggesting low but continuous choline losses [41]. 

In children and adolescents (*N* = 21, 0.75 to 18.6 years), those on home TPN had significantly lower free choline concentrations (6.6 ± 4.3 µmol/L) compared to pediatric surgical patients (*N* = 31) who were enterally fed (8.0 ± 2.3 µmol/L; *p* = 0.02). Whereas plasma choline values were constant in enterally fed children (0.75–18.6 years), they decreased by 0.03µmol/L per month during long-term TPN [42].

Buchman et al. published reference values in relation to age [43], showing higher plasma choline levels in newborns (48 ± 15 µmol/L) that decreased to 12.8 ± 2.0 µmol/L at 3 years and 8.4 ± 3.1 µmol/L in adults. Plasma choline values in newborns were associated with ethnicity and/or socio-economic status (28 ± 13 µmol/L for underprivileged African/Hispanic; 68 ± 17 µmol/L for white middle-class). By contrast, lipid-bound choline increased from 1063 ± 268 µmol/L at birth to 1704 ± 364 µmol/L at 3 years, compared to 2592 ± 584 µmol/L in adults. 

In adult long-term TPN patients [44], deficiency in choline was associated with lack of vitamin B12. Notably, in spite of high folate levels, methylmalonic acid, an indicator of B12-deficiency, was increased in patients with very low plasma choline concentrations. 

The increased risk of catheter thrombosis reported in choline-deficient adult long-term TPN patients [45], may be attributable to increased homocysteine levels resulting from its impaired methylation to methionine which requires betaine (the oxidation product of choline).

In infants born at 28–32 weeks gestation and studied at 4–18 weeks postnatal age, whole blood phosphocholine and glycerophosphocholine (the latter two characteristic intracellular PC precursors and metabolites), rather than plasma choline, tended to be decreased in TPN infants compared to enterally fed controls, whereas TPN lipid administration increased PC [46].

Concerning plasma concentrations of choline and its water-soluble metabolites [18], parturients had higher median choline concentrations (14.1 [10.3–16.9] µmol/L) than non-pregnant women (8.8 [5.7–11.2] µmol/L), but lower betaine and dimethylglycine (11.2 [9.5–14.1] vs. 25.4 [19.2–38.7] µmol/L and 4.4 [3.5–5.9] vs. 6.3 [4.5–8.2] µmol/L, respectively). All values were much higher in cord plasma (41.4 [31.8–51.2], 25.0 [18.2–34.5] and 9.7 [7.8–12.6] µmol/L, respectively), with the highest choline cord plasma concentrations at birth before 32 weeks postmenstrual age (PMA). In preterm infants, plasma concentrations of choline, but not of betaine and dimethylglycine, dropped by ~50% (21.5 [16.4–28.4] µmol/L) within 48h of birth.

Finally, in stable preterm infants <27 weeks PMA [47], D_9_-choline incorporation into plasma PC, as a proxy of de novo PC synthesis from exogenous choline, was 2.5-fold higher than in adults (1.26% vs. 0.51%), reaching peak values at 12 rather than 24h with a plasma half-life of ~72 h rather than ~148 h, suggesting a higher rate of PC synthesis and accelerated turnover. Preterm infants’ PC synthesis via the methylation of phosphatidylethanolamine (PE) by PEMT, however, was only 4.0% of de novo synthesis from exogenous choline, compared to 63% in adults.

### 3.4. Interventional Studies in Humans

Tayek et al. [49] compared TPN with parenteral lipids (*N* = 12, supplying 73 µg free choline, i.e., 0.01% of AI and 241mg PC-choline (43.8% of AI) per day) and TPN without lipids and choline in 13 malnourished adults (19–90 years). The lipid group showed a strong increase in AST, peaking at d7, and liver impairment progressing with the duration of TPN.

In a randomized, double-blind placebo-controlled trial in 15 stable adult long-term (1.5–12.5 years) TPN patients receiving 22 ± 5% of energy via intravenous lipids [50], PC was supplemented orally (2 × 20 g/d, equaling 5.6 g choline, i.e., 10fold AI). The PC group showed a rapid increase in plasma choline within 2 weeks that was sustained throughout the 6 weeks study period. The authors reported a significant and progressive decrease in liver fat in the enteral PC group.

In a pilot study [52], 1–4 g/d choline chloride was injected into the parenteral lipid emulsion and administered IV over 10–12 h/d in 4 long-term TPN patients (43–72 years). Initial plasma choline (2.7–7.2 µmol/L vs. 11.4 ± 3.7 µmol/L in 8 controls, 22–34 years) increased to high-normal values within 1 week, but rapidly returned to low levels after discontinuation of choline administration. Normalization of steatosis was evident during administration (2–6 weeks), lasted for 4 weeks after the end of administration (*N* = 4), but relapsed 10 weeks later (*N* = 1). Liver enzymes either remained constant or decreased. However, if liver enzymes decreased with choline, they increased again with choline discontinuation.

Subsequently, adults (24–55 years) on long-term TPN were randomized to supplementation with 2 g/d choline chloride IV (i.e., 1.49 g choline, the 2.7-fold AI value (*N* = 7)) or placebo (*N* = 8) for 24 weeks with a 10 weeks follow-up [53]. Plasma choline instantly increased to ~20 µmol/L, phospholipids remained unchanged and serum alkaline phosphatase, alanine aminotransferase and aspartate aminotransferase decreased from 2 weeks onwards. Liver steatosis, measured as Houndsfield units, was normalized from 4 weeks onwards throughout supplementation, but relapsed 10 weeks after discontinuation of choline. Additionally, improved verbal and visual memory were reported in supplemented patients [59]. Choline chloride was added as a watery 50% stand-alone solution to the parenteral formulation, prepared by the delivering pharmacy up to 24 weeks prior to application [53,54]. 

An open-label unblinded study [56] examined parenteral choline chloride solutions at age-adjusted AI doses in 7 stable (hepatic or renal failure excluded) term-born (*N* = 5) or preterm (gestational age 26 and 32 weeks) children, aged from 3.2 months to 14 years. Patients predominantly received parenteral nutrition for at least 1 month. Only the two children older than 9 years responded with normalization of plasma choline concentrations (P25–P75 values), whereas in infants (3.2 to 7.9 months), plasma choline remained below the third percentile. One preterm infant (32 weeks gestational age, small intestinal resection following jejunal atresia) suffered from cystic fibrosis, where choline requirements are significantly increased, due to fecal PC losses resulting from exocrine pancreatic insufficiency [74,75,76]. Despite persistently low plasma choline in infants, increased transaminases tended to decrease following start of choline supplementation, but did not normalize at this dose.

### 3.5. Reviews

The identified reviews [27,58,59,60,61,62,63,64,65,66,67,68,69,70,71,72,73] highlighted the following main issues in the broader context of choline administration during TPN. Of these, refs. [27,59,60,62,63,64,65,67,68,69,70,71,72,73] referred to humans, while refs. [58,61,68] referred to mammalian choline metabolism including human liver disease in vivo. Ref. [66] addressed the respective mouse models for choline deficiency-related liver disease, while ref. [73] specifically did so for the function of human cystathionine gamma-lyase being central to hepatic choline and transsulfuration pathways.

Choline is essential for PC, SPH and acetylcholine synthesis and, via oxidation to betaine, as a methyl donor [58].

Choline deficiency results in liver damage and deteriorated secretion of VLDL in various vertebrate animal models and humans. Hepatic steatosis during TPN and late pregnancy in part indicates choline deficiency. Severity of steatosis and increase in liver enzymes during TPN are inversely related to choline plasma concentrations. Experimental choline deficiency induces liver pathology more rapidly than other alimentary factors (excess fat, excess sucrose/fructose) [58,59,63,64,66].

Intestinal failure or parenteral nutrition-associated liver disease (IFALD, PNALD) is dominated by steatosis in adults, but by cholestasis in children. It has a complex pathophysiology including immaturity, sepsis, impaired enterohepatic cycle function, intestinal hormone and bile secretion, and contributions of excessive macronutrient supply together with deficiencies in choline, cysteine, glutathione, and taurine [61,62,65].

Deficiency in (free) choline in PN-preparations results in low plasma choline concentration, which cannot be compensated by lipid-bound choline in lipid emulsions [57,58,59,63].

Choline metabolism is linked to methyl-tetrahydrofolate, vitamin B_12_ and methionine metabolism [58].

Methionine largely bypasses the liver during PN, prohibiting sufficient synthesis of cysteine and taurine from methionine via the transsulfuration pathway. Low plasma choline is associated with high homocysteine concentrations [57,68] (see Figure 2). 

Betaine, the hepatic oxidation product of choline, is required as a methyl donor for S-adenosyl-methionine (SAM) formation for cysteine synthesis from homocysteine in a vitamin B6-dependent reaction, followed by taurine and glutathione formation from cysteine [58,72,73].

In adults, choline administered enterally (2.5–5.5 g/d) or parenterally via choline chloride infusion (1100 mg/d) [58], is suggested to compensate for insufficient choline supply during TPN.

Intestinal microbiota and single nucleotide polymorphisms impairing endogenous choline synthesis should be addressed with respect to the pathogenesis of choline deficiency during TPN, where it causes impaired diversity and a shift towards Firmicuta and opportunistic pathogenic bacteria. While mutations in choline metabolism and transporters, like SLC44A1, are rare, those in PEMT are more frequent [64,67,68].

The association of enteral choline intake with atherosclerosis via bacterial formation of trimethylamine (TMA) in the intestine that is further metabolized to TMA oxide (TMAO) in the liver [68].

Choline deficiency may already be present even before choline-free TPN is started, due to maternal nutrition and PEMT genetics, the degree of immaturity and current feeding strategies. It may be a key factor in other intestinal complications as well, like necrotizing enterocolitis in preterm infants [27].

### 3.6. Guidelines

In 2012 and 2015, the American Society of Parenteral and Enteral Nutrition (A.S.P.E.N.) published a position paper [23,69] calling for the inclusion of free choline, either as a stand-alone product or as part of a multi-component fortifier, to provide TPN patients with free choline supplying the adequate intake (AI) as defined by the National Academy of Medicine (NAM), the former Institute of Medicine (IOM) [20]. 

Contrasting preceding positions of the European Society for Paediatric Gastroenterology Hepatology and Nutrition (ESPGHAN), Chinese Society of Parenteral and Enteral Nutrition (CSPEN) and European Society for Clinical Nutrition and Metabolism (ESPEN), ignoring the relevance of choline for parenteral nutrition [23,24,25], a 2022 ESPEN micronutrient guideline [70] defined choline as (1) essential because of insufficient endogenous synthesis, (2) required for structural lipid and acetylcholine synthesis, and (3) important for the prevention of liver steatosis, increased liver enzymes in plasma as well as muscle damage as a consequence of choline deficiency. Furthermore, ESPEN pointed to the impact of adequate choline intake during pregnancy for the prevention of impaired neurological outcome, which is in line with a recent EFSA opinion on fetal and postnatal development 2023 [22].

## 4. Discussion

In this narrative review based on a systematic literature search, we describe preclinical as well as human observational and interventional clinical data on choline deprivation and supplementation during parenteral nutrition, and its potential role in PNALD/IFALD. Notably, interventional data on preterm and term infants as well as older children are virtually absent, although essential nutrient deficiencies likely impact even more on the developing child than later in life. We identified 11 critical topics concerning choline needs in patients undergoing TPN: 

Major differences in choline metabolism between pre-clinical and human studies originate from the faster choline and PC turnover and higher expression of the PEMT pathway in small rodents and the human patients’ higher genetic heterogeneity [14,15,47,77,78]. In mice, liver failure due to choline deficiency can only be achieved by PEMT knockout or combined methionine and choline deficiency [79]. In adult humans, choline intake below adequate intake values (covering the requirements in 75–90% of the population [20,21]) or poor assimilation frequently causes liver disease, while susceptibility depends on individual PEMT expression [80].

Due to low choline administration after birth compared to in utero, plasma choline concentrations rapidly decrease after delivery, i.e., untimely in preterm infants, by ~50% (from ~40 to 20 µmol/L) and are particularly low in preterm infants on TPN [18]. This may impair these patients’ hepatic and other organs’ choline homeostasis, because choline uptake into cells (for PC synthesis) and mitochondria (for betaine synthesis) is proportional to plasma concentrations of free choline [8,9]. Plasma PC concentrations, rapidly increasing after (preterm) delivery, do not reflect choline status [31,46], but may indicate impaired liver function if reduced [11,53].

Normal choline plasma concentrations in relation to age have been established and are highest in the fetus (~40 µmol/L), followed by term infants (~12–15 µmol/L), pregnant women and male adults (~8–12 µmol/L) [18,42]. They continuously decrease in preterm infants in relation to the extent and duration of TPN [48]. Along with this, the adequate intake values to achieve and maintain physiologic plasma choline concentrations amount to ~55 mg/kg/d in preterm infants compared to AI values of 17–18 mg/kg/d in term infants and 7–8 mg/kg/d in adults [13,20,21]. Enteral (preterm) infant nutrition via breast milk achieves a median supply of ~20 mg/kg/d, with large variation plus ~11 mg/kg/d via fortifiers, while formula provides 15–60 mg/kg/d, depending on the product used [81,82]. As physiologic plasma concentrations are proportional to age and growth rate [13,43], low plasma choline concentrations may more rapidly impact on (preterm) infants and children than on older TPN patients.

Generally, parenteral choline has been safe in adults at doses up to 4 times the AI [31,52,53], in line with its upper tolerable limit (UL) data, ranging from 1 g at 1–3 years to 3.5 g in adults, i.e., 6- to 9-times higher than AI values [30]. Notably, parenteral choline has to be given continuously [21,31], as its plasma half-life is very short (~1 h) [14,74,83]. In human nutrition, several choline components are known, i.e., phosphocholine and glycerophosphocholine dominating in milk and PC/SPH in meat and fish. However, in plasma the only water-soluble compound is free choline [18]. While PC is an adequate enteral choline source [50], it does not contribute to choline homeostasis parenterally (see above). Only choline salts, particularly choline chloride, have been tested and may be adequate for parenteral administration [26,31,53,63].

PC synthesis via the PEMT pathway does not even meet choline requirements in adults [11,84]. In preterm infants, endogenous choline synthesis via PEMT is virtually absent, and the transsulfuration pathways linked to SAM is low, impacting on choline (as well as cysteine and taurine) requirements [18,65,84]. Consequently, preterm infants may be prone to a more rapid development of clinical (hepatic) symptoms of choline deficiency than adult TPN patients [13,14,15,47,52]. 

The daily hepatic turnover of PC (via bile [85] and VLDL) [47,83,86] is high. Bile PC must be recovered as lyso-PC, which depends on pancreatic phospholipase A2IB (sPLA2IB), in infants additionally on pancreatic lipase-related protein 2 (PLRP2), and absorption in the terminal ileum [48]. Therefore, enteral choline losses must be expected in patients with enterostomy/short-bowel-syndrome (particularly with small intestinal bacterial overgrowth increasing intestinal choline degradation) and in cystic fibrosis [46,74,85]. Notably, in addition to the lack of choline supply, continuing choline losses via biliary PC secretion may further aggravate choline deficiency in TPN patients with enterostomies [41].

The clinical manifestations of PNALD/IFALD are age-dependent (steatosis in adults, cholestasis in infants) [61] but choline deficiency likely is a contributor in both [50,51,52,87]. Factors such as age, sex, health, growth, pre-existing stores, ongoing losses, and genetic polymorphisms linked to choline metabolism likely impact on resilience and choline requirements [80,88,89,90,91].

The required daily dose for repletion of the hepatic and overall choline pool, once symptomatic choline deficiency has developed in TPN patients, may be higher than established adequate intake (AI) values. In adults, an infusion of 2 g choline chloride (i.e., the 2.7–3.7-fold AI) successfully resolved liver steatosis without adverse effects. In infants, a parenteral AI dose did not normalize plasma concentrations [56]. The AI in preterm infants is not well established, but may be as high as 50–60 mg/kg/d [14,18,92].

While the causality between choline deficiency and PNALD/IFALD is known for 40 years, the number of patients parenterally supplemented with choline in interventional studies is too low to draw conclusions on safety and efficacy. To date, TPN solutions and emulsions are virtually devoid of free choline, in spite of the knowledge that choline is essential for liver homeostasis, growth and prevention or resolution of PNALD/IFALD [51,52,53].

While choline is constitutive in the form of PC and SPH, with tightly regulated concentrations in tissues and secretions [13], its metabolism is linked to that of other components, particularly vitamins B6 (pyridoxin), B9 (folate) and B12 (cobalamin), and components of the transsulfuration pathway (cysteine, methionine) [32] (Figure 2). Deficiencies or abundance of these components may well impact on the clinical relevance of nutritional choline deficiency [44]. These aspects should be included in the design of clinical trials. Moreover, while choline deficiency in TPN patients may exert an acute impact on liver homeostasis and function, initial data on improved cerebral function by choline administration to TPN patients [54] suggests that long-term neurological outcome may be improved by choline administration to pediatric TPN patients, too.

**Figure 3:**
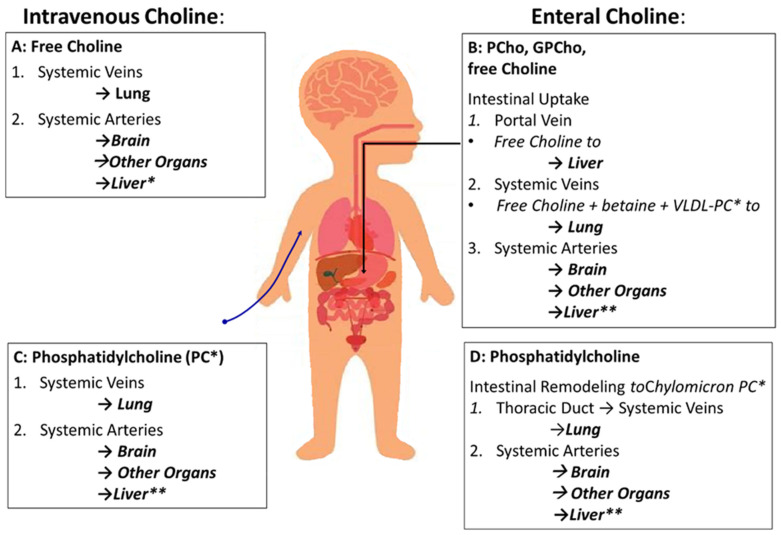
Sequence of organs supplied with choline depending on route of administration (enteral vs. intravenous) and components (water-soluble vs. phosphatidylcholine [PC]). (**A**) Parenteral supply with free choline will supply the lungs first, followed by arterial supply of all other organs via the systemic arteries, including the intestine and liver. (**B**) Enteral supply with water-soluble choline components (free choline [choline chloride] and esters like glycerophosphocholine [GPCho] and phosphocholine [PCho]). Compounds leave the intestine via the portal circulation and enter the liver followed by hepatic secretion of free choline and its metabolites VLDL-PC (20% PC) and betaine. (**C**) Intravenous PC, used as a triglyceride emulsifier, follows the same sequence of organs as intravenous free choline, first passing the lungs followed by the other organs. This route is similar to that of enteral PC administration (**D**), with the exception to enterocytic remodeling of PC followed by its integration into chylomicrons (8% PC) and release into the venous circulation via the thoracic duct. Abbreviations: *, For simplification, sphingomyelin as a minor choline-containing phospholipid in hepatic (VLDL) and intestinal (chylomicrons) secretions was omitted; **, While the liver receives 30% of left ventricular output via the circulation, only ¼ enters the liver directly via the hepatic artery, while ¾ first pass the intestine and spleen and reach the liver via the portal vein.

The sequence of choline supply to organs is different for parenteral compared to enteral supply (Figure 3). The impact of choline compound and route defines that PC in TPN formulations is inefficient to supply the liver and prevent PNALD/IFALD [40].

## 5. Conclusions

While there is a lot of preclinical, observational and interventional clinical and physiological evidence on choline deficiency during TPN, and its contribution to PNALD, IFALD and other conditions, prospective randomized, placebo-controlled trials are missing. These are urgently needed, particularly in infants and children, who have higher choline requirements, and for whom choline may be essential for normal organ growth and long-term development and health.

## Supplementary Materials

The following supporting information can be downloaded at: https://www.mdpi.com/article/10.3390/nu16121873/s1, Table S1: Complete Pubmed searches on the terms “choline” and “parenteral nutrition”.
