# Supplementary material for: Evidence and Perspectives for Choline Supplementation during Parenteral Nutrition—A Narrative Review
Source: Nutrients. 2024 Jun 14;16(12):1873. doi: 10.3390/nu16121873 (PMC11206924; doi:10.3390/nu16121873)
Supplement: Supplementary file 1 [file nutrients-16-01873-s001.zip › nutrients-3017798-supplementary.pdf]

Table S1: Complete Pubmed searches on the terms "choline" and "parenteral nutrition". Data are ordered as appeared in Pubmed searches on January 5, 2023 and April 24, 2024. Selected references are in bold letters.

- January 5; 2023 and April 24; 2024

1: **Berger, M.M.; Shenkin, A.; Schweinlin, A.; Amrein, K.; Augsburger, M.; Biesalski, H.K.; Bischoff; S.C., Casaer, M.P.; Gundogan, K.; Lepp, H.L.; de Man, A.M.E.; Muscogiuri, G.; Pietka, M.; Pironi, L.; Rezzi, S.; Cuerda, C. ESPEN micronutrient guideline. Clin Nutr. 2022, 41, 1357-1424. doi: 10.1016/j.clnu.2022.02.015. Epub 2022 Feb 26. PMID:35365361.**

2: **Corbin, K.D.; Zeisel, S.H. Choline metabolism provides novel insights into nonalcoholic fatty liver disease and its progression. Curr Opin Gastroenterol. 2012, 28, 159-165. doi: 10.1097/MOG.0b013e32834e7b4b. PMID: 22134222; PMCID: PMC3601486.**

3: **Wortmann, S.B.; Mayr, J.A. Choline-related-inherited metabolic diseases-A mini review. J Inherit Metab Dis. 2019, 42, 237-242. doi: 10.1002/jimd.12011. Epub 2019 Jan 25. Erratum in: J Inherit Metab Dis. 2020 Jan;43(1):156. PMID: 30681159; PMCID: PMC7814885.**

4: Bernhard, W.; Poets, C.F.; Franz, A.R. Choline and choline-related nutrients in regular and preterm infant growth. Eur J Nutr. 2019, 58, 931-945. doi: 10.1007/s00394-018-1834-7. Epub 2018 Oct 8. PMID: 30298207.

5: **Vanek, V.W.; Borum, P.; Buchman, A.; Fessler, T.A.; Howard, L.; Jeejeebhoy, K.; Kochevar, M.; Shenkin, A.; Valentine, C.J.; Novel Nutrient Task Force; Parenteral Multi-Vitamin and Multi–Trace Element Working Group; American Society for Parenteral and Enteral Nutrition (A.S.P.E.N.) Board of Directors. A.S.P.E.N. position paper: recommendations for changes in commercially available parenteral multivitamin and multi-trace element products. Nutr Clin Pract. 2012, 27, 440-91. doi: 10.1177/0884533612446706. Epub 2012 Jun 22. Erratum in: Nutr Clin Pract. 2014 Oct;29(5):701. Dosage error in article text. PMID: 22730042.**

6: **Xu, Z.W.; Li, Y.S. Pathogenesis and treatment of parenteral nutrition-associated liver disease. Hepatobiliary Pancreat Dis Int. 2012, 11, 586-593. doi: 10.1016/s1499-3872(12)60229-x. PMID: 23232629.**

7: **Buchman, A.L. The addition of choline to parenteral nutrition. Gastroenterology. 2009, 137(5 Suppl), S119-S128. doi:10.1053/j.gastro.2009.08.010. PMID: 19874943.**

8: **Nilsson, A.K.; Pedersen, A.; Malmodin, D.; Lund, A.M.; Hellgren, G.; Löfqvist, C.; Pupp, I.H.; Hellström, A. Serum choline in extremely preterm infants declines with increasing parenteral nutrition. Eur J Nutr. 2021, 60, 1081-1089. doi:10.1007/s00394-020-02312-2. Epub 2020 Jun 25. PMID: 32588218; PMCID: PMC7900091.**

9: **Cahova, M.; Bratova, M.; Wohl, P. Parenteral Nutrition-Associated Liver Disease: The Role of the Gut Microbiota. Nutrients. 2017, 9, 987. doi:10.3390/nu9090987. PMID: 28880224; PMCID: PMC5622747.**

10: **Zhu, J.; Wu, Y.; Guo, Y.; Tang, Q.; Lu, T.; Cai, W.; Huang, H. Choline Alleviates Parenteral Nutrition-Associated Duodenal Motility Disorder in Infant Rats. JPEN J Parenter Enteral Nutr. 2016, 40, 995-1005. doi: 10.1177/0148607115583674. Epub 2015 Apr 22. PMID: 25904588.**

11: **Sentongo, T.A.; Kumar, P.; Karza, K.; Keys, L.; Iyer, K.; Buchman, A.L. Whole-blood-free choline and choline metabolites in infants who require chronic parenteral nutrition therapy. J Pediatr Gastroenterol Nutr. 2010, 50, 194-199. doi: 10.1097/MPG.0b013e3181a93735. PMID: 20038853.**

12: Cashman, J.R.; Lattard, V.; Lin, J. Effect of total parenteral nutrition and choline on hepatic flavin-containing and cytochrome P-450 monooxygenase activity in rats. Drug Metab Dispos. 2004, 32, 222-229. doi: 10.1124/dmd.32.2.222. PMID: 14744944.

13: **Zhu, J.; Lu, T.; Chen, F.; Yan, J.; Chen, F.; Zhang, Q.; Wang, J.; Yan, W.; Yu, T.; Tang, Q.; Cai, W. Choline Protects Against Intestinal Failure-Associated Liver Disease in Parenteral Nutrition-Fed Immature Rats. JPEN J Parenter Enteral Nutr. 2018, 42, 436-445. doi: 10.1177/0148607116677048. Epub 2017 Dec 15. Erratum in: JPEN J Parenter Enteral Nutr. 2018, 42, 490. PMID: 27856995.**

14: **Kumpf, V.J. Parenteral nutrition-associated liver disease in adult and pediatric patients. Nutr Clin Pract. 2006, 21, 279-90. doi: 10.1177/0115426506021003279. PMID: 16772545.**

15: **Compher, C.W.; Kinosian, B.P.; Stoner, N.E.; Lentine, D.C.; Buzby, G.P. Choline and vitamin B12 deficiencies are interrelated in folate-replete long-term total parenteral nutrition patients. JPEN J Parenter Enteral Nutr. 2002, 26, 57-62. doi: 10.1177/014860710202600157. PMID: 11833752.**

16: Teran, J.C. Nutrition and liver diseases. Curr Gastroenterol Rep. 1999, 1, 335-340. doi: 10.1007/s11894-999-0119-y. PMID: 10980970.

17: **Buchman, A.L.; Sohel, M.; Brown, M.; Jenden, D.J.; Ahn, C.; Roch, M.; Brawley, T.L. Verbal and visual memory improve after choline supplementation in long-term total parenteral nutrition: a pilot study. JPEN J Parenter Enteral Nutr. 2001, 25, 30-35. doi: 10.1177/014860710102500130. PMID: 11190987.**

18: Moreno Villares, J.M. Complicaciones hepáticas asociadas al uso de nutrición parenteral [Parenteral nutrition-associated liver disease]. Nutr Hosp. 2008, 23 Suppl 2, 25-33. Spanish. PMID: 18714408.

19: Gabe, S.M.; Culkin, A. Abnormal liver function tests in the parenteral nutrition fed patient. Frontline Gastroenterol. 2010, 1, 98-104. doi:10.1136/fg.2009.000521. Epub 2010 Jun 15. PMID: 28839556; PMCID: PMC5536778.

20: **Shronts, E.P. Essential nature of choline with implications for total parenteral nutrition. J Am Diet Assoc. 1997, 97, 639-646; 649; quiz 647-8. doi: 10.1016/S0002-8223(97)00161-2. PMID: 9183326.**

21: Lloyd DA; Gabe SM. Managing liver dysfunction in parenteral nutrition. Proc Nutr Soc. 2007, 66, 530-538. doi: 10.1017/S002966510700585X. PMID: 17961274.

22: **Buchman AL; Moukarzel A; Jenden DJ; Roch M; Rice K; Ament ME. Low plasma free choline is prevalent in patients receiving long term parenteral nutrition and is associated with hepatic aminotransferase abnormalities. Clin Nutr. 1993, 12, 33-37. doi: 10.1016/0261-5614(93)90143-r. PMID: 16843274.**

23: **Buchman, A.L.; Sohel, M.; Moukarzel, A.; Bryant, D.; Schanler, R.; Awal, M.; Burns, P.; Dorman, K.; Belfort, M.; Jenden, D.J.; Killip, D.; Roch, M. Plasma choline in normal newborns; infants; toddlers; and in very-low-birth-weight neonates requiring total parenteral nutrition. Nutrition. 2001, 17, 18-21. doi: 10.1016/s0899-9007(00)00472-x. PMID: 11165882.**

24: Shunova, A.; Böckmann, K.A.; Minarski, M.; Franz, A.R.; Wiechers, C.; Poets, C.F.; Bernhard, W. Choline Content of Term and Preterm Infant Formulae Compared to Expressed Breast Milk-How Do We Justify the Discrepancies? Nutrients. 2020, 12, 3815. doi: 10.3390/nu12123815. PMID: 33322176; PMCID: PMC7763895.

25: **Buchman, A.L.; Dubin, M.; Jenden, D.; Moukarzel, A.; Roch, M.H.; Rice, K.; Gornbein, J.; Ament, M.E.; Eckhert, C.D. Lecithin increases plasma free choline and decreases hepatic steatosis in long-term total parenteral nutrition patients. Gastroenterology. 1992, 102, 1363-1370. PMID: 1551541.**

26: **Misra, S.; Ahn, C.; Ament, M.E.; Choi, H.J.; Jenden, D.J.; Roch, M.; Buchman, A.L. Plasma choline concentrations in children requiring long-term home parenteral nutrition: a case control study. JPEN J Parenter Enteral Nutr. 1999, 23, 305-308. doi: 10.1177/0148607199023005305. PMID: 10485444.**

27: Sokol, R.J.; Taylor, S.F.; Devereaux, M.W.; Khandwala, R.; Sondheimer, N.J.; Shikes, R.H.; Mierau, G. Hepatic oxidant injury and glutathione depletion during total parenteral nutrition in weanling rats. Am J Physiol. 1996, 270, G691-700. doi: 10.1152/ajpgi.1996.270.4.G691. PMID: 8928800.

28: **Kitchen, P.; Forbes, A. Parenteral nutrition. Curr Opin Gastroenterol. 2003, 19, 144-7. doi: 10.1097/00001574-200303000-00008. PMID: 15703555.**

29: **Buchman, A.L.; Dubin, M.D.; Moukarzel, A.A.; Jenden, D.J.; Roch, M.; Rice, K.M.; Gornbein, J.; Ament, M.E. Choline deficiency: a cause of hepatic steatosis during parenteral nutrition that can be reversed with intravenous choline supplementation. Hepatology. 1995, 22, 1399-1403. PMID: 7590654.**

30: Young, R.C.; Blass, J.P. Iatrogenic nutritional deficiencies. Annu Rev Nutr. 1982, 2, 201-227. doi: 10.1146/annurev.nu.02.070182.001221. PMID: 6764730.

31: Martínez, M.; Ballabriga, A. Effects of parenteral nutrition with high doses of linoleate on the developing human liver and brain. Lipids. 1987, 22, 133-138. doi: 10.1007/BF02537290. PMID: 3106740.

32: Yang, H.; Finaly, R.; Teitelbaum, D.H. Alteration in epithelial permeability and ion transport in a mouse model of total parenteral nutrition. Crit Care Med. 2003, 31, 1118-1125. doi: 10.1097/01.CCM.0000053523.73064.8A. PMID: 12682482.

33: **Buchman, A.L.; Ament, M.E.; Sohel, M.; Dubin, M.; Jenden, D.J.; Roch, M.; Pownall, H.; Farley, W.; Awal, M.; Ahn, C. Choline deficiency causes reversible hepatic abnormalities in patients receiving parenteral nutrition: proof of a human choline requirement: a placebo-controlled trial. JPEN J Parenter Enteral Nutr. 2001, 25, 260-268. doi: 10.1177/0148607101025005260. PMID: 11531217.**

34: **Goss, K.C.W.; Goss, V.M.; Townsend, J.P.; Koster, G.; Clark, H.W.; Postle, A.D. Postnatal adaptations of phosphatidylcholine metabolism in extremely preterm infants: implications for choline and PUFA metabolism. Am J Clin Nutr. 2020, 112, 1438-1447. doi: 10.1093/ajcn/nqaa207. PMID: 32778895; PMCID:PMC7727469.**

35: **da Costa, K.A.; Niculescu, M.D.; Craciunescu, C.N.; Fischer, L.M.; Zeisel, S.H. Choline deficiency increases lymphocyte apoptosis and DNA damage in humans. Am J Clin Nutr. 2006, 84, 88-94. doi: 10.1093/ajcn/84.1.88. PMID: 16825685; PMCID: PMC2430662.**

36: **Buchman, A.L. Choline deficiency during parenteral nutrition in humans. Nutr Clin Pract. 2003, 18, 353-358. doi: 10.1177/0115426503018005353. PMID: 16215064.**

37: Buchman, A.L.; Jenden, D.; Suki, W.N.; Roch, M. Changes in plasma free and phospholipid-bound choline concentrations in chronic hemodialysis patients. J Ren Nutr. 2000, 10, 133-138. doi: 10.1053/jren.2000.7411. PMID: 10921534.

38: **Kelly, D.A. Intestinal failure-associated liver disease: what do we know today? Gastroenterology. 2006, 130(2 Suppl 1), S70-S77. doi: 10.1053/j.gastro.2005.10.066. PMID: 16473076.**

39: **Wan, S.; Yang, J.; Mamtawla, G.; Zhang, L.; Gao, X.; Wang, X. Differential Metabolomic Analysis of Liver Tissues from Rat Models of Parenteral Nutrition-Associated Liver Disease. Biomed Res Int. 2020, 9156359. doi: 10.1155/2020/9156359. PMID: 32280707; PMCID: PMC7115143.**

40: **Burt, M.E.; Hanin, I.; Brennan, M.F. Choline deficiency associated with total parenteral nutrition. Lancet. 1980, 2(8195 pt 1) , 638-639. doi: 10.1016/s0140-6736(80)90301-3. PMID: 6107423.**

41: Woodward, J.M.; Priest, A.N.; Hollingsworth, K.G.; Lomas, D.J. Clinical application of magnetic resonance spectroscopy of the liver in patients receiving long-term parenteral nutrition. JPEN J Parenter Enteral Nutr. 2009, 33, 669-676. doi: 10.1177/0148607109332908. Epub 2009 Jul 8. PMID: 19587386.

42: Müller, M.J. Hepatische Komplikationen bei parenteraler Ernährung [Hepatic complications in parenteral nutrition]. Z Gastroenterol. 1996, 34, 36-40. German. PMID: 8776174.

43: **Rudman, D.; Williams, P.J. Nutrient deficiencies during total parenteral nutrition. Nutr Rev. 1985, 43, 1-13. doi: 10.1111/j.1753-4887.1985.tb02383.x. PMID: 3920582.**

44: **Demetriou, A.A. Lecithin increases plasma free choline and decreases hepatic steatosis in long-term total parenteral nutrition patients. JPEN J Parenter Enteral Nutr. 1992, 16, 487-488. doi: 10.1177/0148607192016005487. PMID:1433785.**

45: Poklis, J.L.; Mohs, A.J.; Wolf, C.E.; Poklis, A.; Peace, M.R. Identification of Drugs in Parenteral Pharmaceutical Preparations from a Quality Assurance and a Diversion Program by Direct Analysis in Real-Time AccuTOFTM-Mass Spectrometry (DART-MS). J Anal Toxicol. 2016, 40, 608-616. doi: 10.1093/jat/bkw065. Epub 2016 Jul 29.PMID: 27474362; PMCID: PMC5048709.

46: **Maherzi, A.; Vatier, J.; Cezard, J.P.; Ferkdadji, L.; Duet, M.; Celice-Pinguaud, C.; Peuchmaur, M.; Navarro, J. Characteristics and consequences of duodenogastric reflux in children on total parenteral nutrition (TPN) for severe gastrointestinal disorders. Clin Nutr. 1994, 13, 345-350. doi: 10.1016/0261-5614(94)90023-x. PMID: 16843412.**

47: Hyde, M.J.; Amusquivar, E.; Laws, J.; Corson, A.M.; Geering, R.R.; Lean, I.J.; Putet, G.; Dodds, P.F.; Herrera, E.; Clarke, L. Effects of lipid-supplemented total parenteral nutrition on fatty liver disease in a premature neonatal piglet model. Neonatology. 2008, 93, 77-86. doi: 10.1159/000106784. Epub 2007 Aug 6. PMID: 17684422.

48: Puntis, J.W. Nutritional support at home and in the community. Arch Dis Child. 2001, 84, 295-8. doi: 10.1136/adc.84.4.295. PMID: 11259223; PMCID: PMC1718713.

49: **Narkewicz, M.R.; Caldwell, S.; Jones, G. Cysteine supplementation and reduction of total parenteral nutrition-induced hepatic lipid accumulation in the weanling rat. J Pediatr Gastroenterol Nutr. 1995, 21, 18-24. doi: 10.1097/00005176-199507000-00003. PMID: 8576809.**

50: **Yan, J.K.; Zhu, J.; Gong, Z.Z.; Wen, J.; Xiao, Y.T.; Zhang, T.; Cai, W. Supplementary choline attenuates olive oil lipid emulsion-induced enterocyte apoptosis through suppression of CELF1/AIF pathway. J Cell Mol Med. 2018, 22, 1562-1573. doi: 10.1111/jcmm.13430. Epub 2017 Nov 6. PMID: 29105957; PMCID: PMC5824412.**

51: Allard, J.P. Other disease associations with non-alcoholic fatty liver disease (NAFLD). Best Pract Res Clin Gastroenterol. 2002, 16, 783-795. doi: 10.1053/bega.2002.0330. PMID: 12406445.

52: **Vanek, V.W.; Borum, P.; Buchman, A.; Fessler, T.A.; Howard, L.; Shenkin, A.; Valentine, C.J.; Novel Nutrient Task Force; Parenteral Vitamin and Trace Element Working Group; and the American Society for Parenteral and Enteral Nutrition (A.S.P.E.N.); Novel Nutrient Task Force Parenteral Vitamin and Trace Element Working Group and the American Society for Parenteral and Enteral Nutrition A S P E N. A Call to Action to Bring Safer Parenteral Micronutrient Products to the U.S. Market. Nutr Clin Pract. 2015, 30, 559-69. doi: 10.1177/0884533615589992. Epub 2015 Jun 25. PMID: 26113560.**

53: Chawla, R.K.; Wolf, D.C.; Kutner, M.H.; Bonkovsky, H.L. Choline may be an essential nutrient in malnourished patients with cirrhosis. Gastroenterology. 1989, 97, 1514-1520. doi: 10.1016/0016-5085(89)90397-1. PMID: 2511054.

54: **Bernhard, W.; Raith, M.; Kunze, R.; Koch, V.; Heni, M.; Maas, C.; Abele, H.; Poets, C.F.; Franz, A.R. Choline concentrations are lower in postnatal plasma of preterm infants than in cord plasma. Eur J Nutr. 2015, 54, 733-741. doi: 10.1007/s00394-014-0751-7. Epub 2014 Aug 23. PMID: 25148882.**

55: **Tayek, J.A.; Bistrian, B.; Sheard, N.F.; Zeisel, S.H.; Blackburn, G.L. Abnormal liver function in malnourished patients receiving total parenteral nutrition: a prospective randomized study. J Am Coll Nutr. 1990, 9, 76-83. doi: 10.1080/07315724.1990.10720353. PMID: 2106545.**

56: **Buchman, A.L.; Ament, M.E.; Jenden, D.J.; Ahn, C. Choline deficiency is associated with increased risk for venous catheter thrombosis. JPEN J Parenter Enteral Nutr. 2006, 30, 317-320. doi: 10.1177/0148607106030004317. PMID: 16804129.**

57: Mayerle, J.; Simon, P.; Kraft, M.; Meister, T.; Lerch, M.M. Internistische Therapie der akuten Pankreatitis [Conservative treatment of acute pancreatitis]. Med Klein (Munich). 2003, 98, 744-749. German. doi: 10.1007/s00063-003-1320-7. PMID: 14685675.

58: **Guerrerio, A.L.; Mattis, L.; Conner, K.G.; Hampsey, J.; Stasinopoulos, D.M.; DeJong, R.; Boctor, E.M;. Sheth, S.; Hamper, U.M.; Scheimann, A.O. Oral choline supplementation in children with intestinal failure. J Pediatr Gastroenterol Nutr. 2011, 53, 115-119. doi: 10.1097/MPG.0b013e31821404d4. PMID: 21694550.**

59: **Buchman, A.L.; Jenden, D.J.; Moukarzel, A.A.; Roch, M.; Rice, K.M.; Chang, A.S.; Ament, M.E. Choline pharmacokinetics during intermittent intravenous choline infusion in human subjects. Clin Pharmacol Ther. 1994, 55, 277-283. doi: 10.1038/clpt.1994.28. PMID: 8143393.**

60: **Sheard, N.F.; Tayek, J.A.; Bistrian, B.R.; Blackburn, G.L.; Zeisel, S.H. Plasma choline concentration in humans fed parenterally. Am J Clin Nutr. 1986, 43, 219-224. doi: 10.1093/ajcn/43.2.219. PMID: 3080867.**

61: Brodin, B.; Cederblad, G.; Larsson, J.; Schildt, B.; Sjödahl, R.; Symreng, T.; Wetterfors, J. Rapid determination of cold insoluble globulin by laser nephelometry. Application in patients receiving preoperative total parenteral nutrition. JPEN J Parenter Enteral Nutr. 1982, 6, 214-217. doi: 10.1177/0148607182006003214. PMID: 6809977.

62: **Reid, D.T.; Eksteen, B. Murine models provide insight to the development of non- alcoholic fatty liver disease. Nutr Res Rev. 2015, 28, 133-142. doi: 10.1017/S0954422415000128. Epub 2015 Oct 23. PMID: 26494024.**

63: Sauls, D.L.; Arnold, E.K.; Bell, C.W.; Allen, J.C.; Hoffman, M. Pro-thrombotic and pro- oxidant effects of diet-induced hyperhomocysteinemia. Thromb Res. 2007, 120, 117-126. doi: 10.1016/j.thromres.2006.08.001. Epub 2006 Sep 18. PMID: 16979225.

64: Sheard, N.F.; Krasin, B. Restricting food intake does not exacerbate the effects of a choline-deficient diet on tissue carnitine concentrations in rats. J Nutr. 1994, 124, 738-743. doi: 10.1093/jn/124.5.738. PMID: 8169667.

65: **Oz, H.S.; Im, H.J.; Chen, T.S.; de Villiers, W.J.; McClain, C.J. Glutathione-enhancing agents protect against steatohepatitis in a dietary model. J Biochem Mol Toxicol. 2006, 20, 39-47. doi: 10.1002/jbt.20109. PMID: 16498637; PMCID: PMC3006092.**

66: Lekka, M.E.; Liokatis, S.; Nathanail, C.; Galani, V.; Nakos, G. The impact of intravenous fat emulsion administration in acute lung injury. Am J Respir Crit Care Med. 2004, 169, 638-644. doi: 10.1164/rccm.200305-620OC. Epub 2003 Dec 4. PMID: 14656749.

67: **Chawla, R.K.; Berry, C.J.; Kutner, M.H.; Rudman, D. Plasma concentrations of transsulfuration pathway products during nasoenteral and intravenous hyperalimentation of malnourished patients. Am J Clin Nutr. 1985, 42, 577-584. doi: 10.1093/ajcn/42.4.577. PMID: 3931450.68: Hager L. Choline deficiency and TPN associated liver dysfunction: a case report. Nutrition. 1998 Jan;14(1):60-2. PMID: 9437687.**

69: **Hall, R.I.; Ross, L.H.; Bozovic, M.G.; Grant, J.P. The effect of choline supplementation on hepatic steatosis in the parenterally fed rat. JPEN J Parenter Enteral Nutr. 1985, 9, 597-599. doi: 10.1177/0148607185009005597. PMID: 3930766.**

70: Joyeux, H.; Joyeux, A.; Raoux, P.; Brissac, C.; Blanc, F.; Solassol, C. Troubles métaboliques de l'anhépatie expérimentale et nutrition parentérale [Metabolic disorders in experimental hepatic insufficiency and parenteral feeding]. Ann Anesthesiol Fr. 1977, 18, 939-947. French. PMID: 24386.

71: **Morán Penco, J.M.; Maciá Botejara, E.; Salas Martinez, J.; Mahedero Ruiz, G.; Climent Mata, V.; Saenz de Santamaria, J.; Vinagre Velasco, L.M. Liver lipid composition and intravenous; intraperitoneal; and enteral administration of intralipid. Nutrition. 1994, 10, 26-31. PMID: 8199419.**

72: Ohni, M.; Hata, Y. [Nutritional assessment and nutritional support therapy in elderly patients]. Nihon Ronen Igakkai Zasshi. 1993, 30, 587-594. Japanese. doi: 10.3143/geriatrics.30.587. PMID: 8361076.

73: Mayer, K.; Fegbeutel, C.; Hattar, K.; Sibelius, U.; Krämer, H.J.; Heuer, K.U.; Temmesfeld-Wollbrück, B.; Gokorsch, S.; Grimminger, F.; Seeger, W. Omega-3 vs. omega-6 lipid emulsions exert differential influence on neutrophils in septic shock patients: impact on plasma fatty acids and lipid mediator generation. Intensive Care Med. 2003, 29, 1472-1481. doi: 10.1007/s00134-003-1900-2. Epub 2003 Jul 25. PMID: 12897994; PMCID: PMC7187949.

74: Stennett, D.J.; Gerwick, W.H.; Egging, P.K.; Christensen, J.M. Precipitate analysis from an indwelling total parenteral nutrition catheter. JPEN J Parenter Enteral Nutr. 1988, 12, 88-92. doi: 10.1177/014860718801200188. PMID: 3125364.

75: Imeryuz, N.; Tahan, V.; Sonsuz, A.; Eren, F.; Uraz, S.; Yuksel, M.; Akpulat, S.; Ozcelik, D.; Haklar, G.; Celikel, C.; Avsar, E.; Tozun, N. Iron preloading aggravates nutritional steatohepatitis in rats by increasing apoptotic cell death. J Hepatol. 2007, 47, 851-859. doi: 10.1016/j.jhep.2007.06.018. Epub 2007 Aug 13. PMID: 17825453.

76: Mau, T.; Eckley, S.S.; Bergin, I.L.; Saund, K.; Villano, J.S.; Vendrov, K.C.; Snitkin, E.S.; Young, V.B.; Yung, R. Outbreak of Murine Infection with <i>Clostridium difficile</i> Associated with the Administration of a Pre- and Perinatal Methyl Donor Diet. mSphere. 2019, 4, e00138-19. doi: 10.1128/mSphereDirect.00138-19. PMID: 30894434; PMCID: PMC6429045.

77: Paymaster, N.J. Postoperative magnesium deficiency. Br J Anaesth. 1975, 47, 85-87. doi: 10.1093/bja/47.1.85. PMID: 807227.

78: Rudman, D.; Feller, A. Evidence for deficiencies of conditionally essential nutrients during total parenteral nutrition. J Am Coll Nutr. 1986, 5, 101-106. doi: 10.1080/07315724.1986.10720117. PMID: 3088080.

79: Bozzetti, F.; Terno, G.; Pupa, A.; Uccellini, M.; Rota, G.; Emanuelli, H. Iperalimentazione parenterale nei pazienti con neoplasia avanzata [Parenteral hyperalimentation in patients with advanced

neoplastic disease (author's transl)]. Tumori. 1976, 62, 623-644. Italian. doi: 10.1177/030089167606200606. PMID: 828982.

80: Wanten, G.; Kusters, A;. van Emst-de Vries, S.E.; Tool, A.; Roos, D.; Naber, T.; Willem,s P. Lipid effects on neutrophil calcium signaling induced by opsonized particles: platelet activating factor is only part of the story. Clin Nutr. 2004, 23, 623-630. doi: 10.1016/j.clnu.2003.10.014. PMID: 15297099.

81: Price, J.B. Jr.; Takeshige, K.; Parsa, M.; Voorhees, A.B. Jr. Maintenance of dogs without splanchnic portal organs. Surg Forum. 1970, 21, 380-381. PMID: 4999014.

82: Tsallas, G.; Baun, D.C. Home care total parenteral alimentation. Am J Hosp Pharm. 1972, 29, 840-846. PMID: 4627575.

83: Nachtwey, W. Zur Behandlung des Wundstarrkrampfes [On The Treatment of Tetanus after Trauma]. Munch Med Wochenschr. 1963, 105, 2285-2287. German. PMID: 14098421.

84: Kaminski, D.L.; Adams, A.; Jellinek, M. The effect of hyperalimentation on hepatic lipid content and lipogenic enzyme activity in rats and man. Surgery. 1980, 88, 93-100. PMID: 6104363.

- April 24; 2024, additional reference compared to January 5; 2023

85. **Drenckpohl, D.C.; Christifano, D.N.; Carlson, S.E. Is choline deficiency an unrecognized factor in necrotizing enterocolitis of preterm infants? Pediatr Res. 2024; 24. doi: 10.1038/s41390-024-03212-5. Online ahead of print**.
